# Blood Levels of IL-Iβ, IL-6, IL-8, TNF-α, and MCP-1 in Pneumoconiosis Patients Exposed to Inorganic Dusts

**DOI:** 10.5487/TR.2009.25.4.217

**Published:** 2009-12-30

**Authors:** Jong Seong Lee, Jae Hoon Shin, Joung Oh Lee, Won-Jeong Lee, Joo-Hwan Hwang, Ji Hong Kim, Byung-Soon Choi

**Affiliations:** 18 Center for Occupational Lung Diseases, KWAMCO, 95, II-dong, Sangnok-gu, Ansan-si, Gyeonggi-do, 426-858 Korea; 28 Ansan Choongang General Hospital, KWAMCO, Ansan, 426-858 Korea

**Keywords:** Cytokine, Lung inflammation, Pneumoconiosis

## Abstract

Inhaled inorganic dusts such as coal can cause inflammation and fibrosis in the lung called pneumoconiosis. Chronic inflammatory process in the lung is associated with various cytokines and reactive oxygen species (ROS) formation. Expression of some cytokines mediates inflammation and leads to tissue damage or fibrosis. The aim of the present study was to compare the levels of blood cytokines interleukin (IL)-1β, IL-6, IL-8, tumor necrosis factor (TNF)-α and monocyte chemoattractant protein (MCP)-1 among 124 subjects (control 38 and pneumoconiosis patient 86) with category of chest x-ray according to International Labor Organization (ILO) classification. The levels of serum IL-8 (*p* = 0.003), TNF-α (*p* = 0.026), and MCP-1 (*p* = 0.010) of pneumoconiosis patients were higher than those of subjects with the control. The level of serum IL-8 in the severe group with the small opacity (ILO category II or III) was higher than that of the control (*p* = 0.035). There was significant correlation between the profusion of radiological findings with small opacity and serum levels of IL-1β (*rho* = 0.218, *p* < 0.05), IL-8 (*rho* = 0.224, *p* < 0.05), TNF-α (*rho* = 0.306, *p* < 0.01), and MCP-1 (*rho* = 0.213, *p* < 0.01). The serum levels of IL-6 and IL-8, however, did not show significant difference between pneumoconiosis patients and the control. There was no significant correlation between serum levels of measured cytokines and other associated variables such as lung function, age, BMI, and exposure period of dusts. Future studies will be required to investigate the cytokine profile that is present in pneumoconiosis patient using lung specific specimens such as bronchoalveolar lavage fluid (BALF), exhaled breath condensate, and lung tissue.

## INTRODUCTION

Among occupational lung diseases, most prevalent diseases are induced by inhalation of dusts such as asbestos, crystalline silica and coal. Inhalation of theses dusts may cause a variety of lung diseases such as progressive massive fibrosis (PMF), chronic alveolitis, emphysema, and coal workers pneumoconiosis (CWP). Fibrosis of tissue resulting from theses dusts may invoke functional damage and irreversible change (Schins and Borm, 1999). Notably, crystalline silica has been classified as class I carcinogen by the International Agency for Research on Cancer (IARC, 1997).

Pneumoconiosis is a lung disease caused by inhaling mine dust. Diagnosis of pneumoconiosis depends on morphological changes by radiological findings and functional change by pulmonary function test. Unfortunately, there is no cure for the damage and current diagnostic findings are only limited fibrosis in the lung, which is usually irreversibly progressive. Once silica threshold has been exceed, silica-induced pulmonary disease may progress without further exposure to silica. Therefore, it is important that research on potential and prospective biomarkers for pneumoconiosis should be carried out before irreversible radiological changes in the lung (Gulumian *et al.*, 2006; Porter *et al.*, 2004). Many researchers have studied the role of mediators such as a various cytokines and reactive oxygen species (ROS) in pulmonary inflammation resulting from mineral dusts (Schins and Borm, 1999).

Cytokines have an effect on various biological events such as inflammation, metabolic mechanism, growth and proliferation of the cell, morphogenesis, fibrosis, and homeostasis (Elias and Zitnik, 1992). Major sources of cytokines in the lung are epithelial cells, endothelial cells, fibroblasts and inflammatory cells. In studies for relationship between pulmonary inflammation and dusts, cytokines have been demonstrated for mediator of a various toxicological and pathological effects. The cytokines associated with coal dust exposure were IL-1, IL-4, IL-6, IL-8, IL-10, IL-11, IL-12, IL-13, TNF-$\alpha$, MCP-1, transforming growth factor-$\beta$, insulin-like growth factor-$\beta$, and platelet derived growth factor (Ates *et al.*, 2008; Griwatz *et al.*, 1994; Griwatz and Seemayer, 1994; Prince *et al.*, 2008; Razzaque and Taguchi, 2003; Vanhee *et al.*, 1995; Weber *et al.*, 1996; Ulkor *et al.*, 2008). Although there were a few reports of the relationship between blood cytokines and radiological findings in Korea, these reports not included the pulmonary function test (PFT) and were also controversy about the validity of the radiological findings.

The objective of this study is to investigate the relationship between blood cytokines (IL-1$\beta$, IL-6, IL-8, TNF-$\alpha$, and MCP-1) as inflammation mediator and pneumoconiosis findings obtained from radiological findings identified by the pneumoconiosis review committee and PFT.

## MATERIALS AND METHODS

***Subjects.*** The study population contained 124 retired male workers exposed to inorganic dust, who had lower criteria level related to liver and kidney function such as aspartate aminotransferase, alanine aminotransferase, gamma-glutamyl transpeptidase, blood urea nitrogen, and creatinine. We carried out collecting of blood and urine, chest x-ray, and PFT under the informed consent from all of subjects. Personal information on age, body weight, height, various personal history (job, smoking status and disease) was obtained by a structured questionnaire. The study was approved from the Research Ethics Committee of our research center.

***Analysis of blood cytokines.*** Serum was centrifuged at 3,000 rpm for 10 min. The samples were stored at -80°C until assay. Analysis of blood cytokines was measured by biochip array (EV 3513, Randox Laboratories Ltd., U.K.) using sandwich & competitive chemiluminescence immunoassay, as previously described (Fitzgerald *et al.*, 2005; Molloy *et al.*, 2005). Detection limits of IL-1$\beta$, IL-6, IL-8, TNF-$\alpha$, and MCP-1 were 0.6, 0.4, 1.5, 2.6, and 2.5 pg/ml, respectively.

***Clinical indices of liver and kidney.*** Because inflammation mediator in the blood is influenced by inflammation of whole body, AST, ALT, and $\gamma$-GT were measured as biochemical indicator for liver, and BUN and creatinine were measured as that for kidney using automated biochemical analyzer (Hitachi 7080, Hitachi, Japan) (Brunzel, 2003; Ingram, 2003).

***PFT.*** PFT was performed in accordance with recommended guideline of ATS/ERS Task Force (Brusasco *et al.*, 2005) using spirometer (Vmax22, SensorMedics, USA). We measured forced vital capacity (FVC), which is the volume delivered during an expiration made as forcefully and completely as possible starting from full inspiration, forced expiratory volume in one second ($FEV_1$), which is the volume delivered in the first second of an FVC maneuver, and $FEV_1$/FVC ratio, and calculated predicted volume by regression equation of Morris *et al.* (1971).

$$\text{predicted volume (L)} = 0.0583 \times \text{height (in)} - 0.025 \times \text{age (yr)} - 4.241$$

The predicted percentages (%) of FVC and $FEV_1$ calculated by following way,

$$\text{\% predicted} = \text{measured volume (L)}/\text{predicted volume (L)} \times 100$$

Test of pulmonary function was performed in the sitting position via closed circuit method, measuring inhaled and exhaled air at the same test cycle. Tests were carried out until gaining 3 adequate data.

***Chest x-ray.*** Radiological findings for pneumoconiosis were performed using digital chest x-ray (Digital Diagnost, Philips, Netherlands). Diagnosis of pneumoconiosis was identified by the pneumoconiosis review committee of Korea Worker's Compensation & Welfare Service, and classifications were categorized in accordance with classification of ILO (2002).

***Statistical analysis.*** The data was analyzed using SPSS 14.0 (SPSS, Chicago, IL, USA). General characteristics and PFT data showed normal distribution, whereas the measured cytokines showed log-normal distribution (Kolmogorov-Smirnov test); therefore the data of cytokines were log-transformed for all of the statistical tests, and the results were expressed as the

**Table 1.** Numbers of each pneumoconiosis category according to the ILO classification

| ILO category | N | % | Profusion (N) |
|---|---|---|---|
| Control | 38 | 30.6 | 0/0 (32); 0/1 (6) |
| Small opacity | 67 | 54.1 | |
| I | 40 | 32.3 | 1/0 (11); 1/1 (22); 1/2 (7) |
| II, III | 27 | 21.8 | 2/1 (11); 2/2 (14); 2/3 (1); 3/2 (1) |
| Large opacity | 19 | 15.3 | A (17); B (2) |

geometric mean (GM) and geometric standard deviation (GSD). We analyzed log-transformed data using One-way analysis of variance (ANOVA) followed by Turkey's HSD comparison or t-test. Pearson's product moment correlation coefficient (*r*) was used to assess the correlations between measured cytokines and the considered groups of subjects except for the x-ray profusions expressed Spearman's rank correlation coefficient (*rho*). A *p*-value of $< 0.05$ (two-tailed) was considered significant for all of the tests.

**Table 2.** General characteristics of the study subjects

| | Control (n=38) | Pneumoconiosis | | *p*-values |
|---|---|---|---|---|
| | | Small opacity (n = 67) | Large opacity (n = 19) | |
| Age (yrs) | 61.2 ± 7.2 | 63.6 ± 7.7 | 66.0 ± 7.5 | 0.065[1] |
| BMI (kg/m$^2$) | 22.4 ± 3.0 | 22.4 ± 2.7 | 21.3 ± 2.4 | 0.300[1] |
| Exposure period (yrs) | 17.8 ± 7.7 | 19.4 ± 7.9 | 17.1 ± 6.9 | 0.408[1] |
| FVC, % predicted | 93.0 ± 12.7 | 92.8 ± 13.8 | 92.2 ± 11.5 | 0.974[1] |
| $FEV_1$, % predicted | 88.4 ± 18.2 | 87.7 ± 20.1 | 84.3 ± 15.8 | 0.727[1] |
| $FEV_1$/FVC ratio (%) | 72.0 ± 1.8 | 71.5 ± 1.9 | 70.8 ± 1.7 | 0.068[1] |
| Smoking, N (%) | | | | |
| Never | 8 (47.1) | 7 (41.2) | 2 (11.8) | 0.115[2] |
| Past | 8 (17.4) | 31 (67.4) | 7 (15.2) | |
| Current | 22 (36.1) | 29 (47.5) | 10 (16.4) | |

Arithmetic mean ± standard deviation
[1]Calculated by ANOVA test
[2]Calculated by $\chi^2$-test

**Table 3.** Concentrations of blood cytokines according to general characteristics

| Characteristics | | N | IL-1β | IL-6 | IL-8 | TNF-α | MCP-1 |
|---|---|---|---|---|---|---|---|
| Age (yrs)[1] | ~49 | 7 | 1.51 (2.54) | 1.15 (2.50) | 17.62 (1.77) | 4.68 (1.45) | 185.4 (1.4) |
| | 50~59 | 30 | 1.30 (2.04) | 1.23 (3.71) | 13.43 (1.55) | 3.74 (1.30) | 188.5 (1.6) |
| | 60~69 | 61 | 1.22 (3.47) | 1.31 (2.42) | 14.82 (1.74) | 3.91 (1.33) | 191.2 (1.5) |
| | 70~ | 26 | 0.86 (2.18) | 1.32 (2.07) | 15.40 (1.67) | 3.84 (1.39) | 177.5 (1.3) |
| | | | $p = 0.379$ | $p = 0.980$ | $p = 0.579$ | $p = 0.333$ | $p = 0.874$ |
| BMI (kg/m$^2$)[2] | 25 ≤ | 22 | 1.11 (3.89) | 1.19 (2.23) | 12.45 (1.71) | 3.77 (1.31) | 159.8 (1.3) |
| | < 25 | 102 | 1.18 (2.61) | 1.30 (2.72) | 15.27 (1.67) | 3.92 (1.35) | 193.8 (1.5) |
| | | | $p = 0.794$ | $p = 0.680$ | $p = 0.095$ | $p = 0.560$ | $p = 0.031$ |
| Exposure period (yrs)[1] | ~9 | 16 | 1.60 (4.96) | 1.15 (2.16) | 13.81 (1.54) | 4.12 (1.31) | 175.2 (1.4) |
| | 10~19 | 51 | 1.22 (2.77) | 1.41 (3.44) | 13.38 (1.51) | 3.87 (1.40) | 200.6 (1.5) |
| | 20~29 | 42 | 0.97 (2.28) | 1.31 (2.14) | 17.01 (1.94) | 3.89 (1.31) | 187.3 (1.4) |
| | 30~ | 15 | 1.19 (2.34) | 0.99 (1.78) | 14.61 (1.50) | 3.73 (1.26) | 159.1 (1.6) |
| | | | $p = 0.417$ | $p = 0.626$ | $p = 0.156$ | $p = 0.817$ | $p = 0.182$ |
| Smoking[1] | Never | 17 | 0.78 (2.03) | 0.96 (2.03) | 13.31 (1.53) | 3.77 (1.27) | 167.4 (1.3) |
| | Past | 46 | 1.12 (3.02) | 1.26 (2.08) | 15.24 (1.69) | 4.19 (1.32) | 183.8 (1.6) |
| | Current | 61 | 1.35 (2.82) | 1.41 (3.19) | 14.77 (1.72) | 3.71 (1.36) | 196.0 (1.4) |
| | | | $p = 0.143$ | $p = 0.339$ | $p = 0.659$ | $p = 0.094$ | $p = 0.298$ |
| %FVC predicted[2] | 80 ≤ | 103 | 1.23 (2.93) | 1.29 (2.82) | 14.46 (1.65) | 3.95 (1.34) | 185.5 (1.5) |
| | < 80 | 21 | 0.91 (2.16) | 1.25 (1.65) | 16.11 (1.83) | 3.61 (1.34) | 196.6 (1.4) |
| | | | $p = 0.223$ | $p = 0.882$ | $p = 0.388$ | $p = 0.198$ | $p = 0.527$ |
| %$FEV_1$ predicted[2] | 80 ≤ | 87 | 1.21 (3.04) | 1.22 (2.87) | 14.16 (1.63) | 3.76 (1.29) | 181.2 (1.5) |
| | < 80 | 37 | 1.08 (2.29) | 1.45 (2.04) | 16.15 (1.78) | 4.23 (1.43) | 202.4 (1.5) |
| | | | $p = 0.566$ | $p = 0.348$ | $p = 0.198$ | $p = 0.072$ | $p = 0.141$ |
| %$FEV_1$/FVC ratio[2] | 70 ≤ | 100 | 1.25 (2.88) | 1.24 (2.53) | 14.69 (1.69) | 3.95 (1.32) | 183.7 (1.4) |
| | < 70 | 24 | 0.88 (2.46) | 1.46 (3.10) | 14.90 (1.64) | 3.64 (1.41) | 202.8 (1.6) |
| | | | $p = 0.136$ | $p = 0.460$ | $p = 0.903$ | $p = 0.222$ | $p = 0.258$ |

Geometric mean (Geometric standard deviation), unit: pg/ml
[1]Calculated by ANOVA test
[2]Calculated by t-test

**Table 4.** Concentrations of IL-1β, IL-6, IL-8, TNF-α and MCP-1 in the blood of pneumoconiosis patients

| Cytokines | | N | GM[1] | GSD | Range | *p*-values of difference[2] | |
|---|---|---|---|---|---|---|---|
| | | | | | | 2 | 3 |
| IL-1β | 1. Control | 38 | 1.24 | 3.16 | ND[3]~201.33 | 1.000 | 1.000 |
| | 2. Small opacity | 67 | 1.14 | 2.77 | ND~68.57 | | 1.000 |
| | 3. Large opacity | 19 | 1.11 | 2.40 | ND~6.66 | | |
| | | | F = 0.105 (*p* = 0.900) | | | | |
| IL-6 | 1. Control | 38 | 1.09 | 2.63 | 0.36~17.41 | 0.900 | 0.750 |
| | 2. Small opacity | 67 | 1.34 | 2.48 | 0.38~193.09 | | 1.000 |
| | 3. Large opacity | 19 | 1.50 | 3.18 | 0.55~74.33 | | |
| | | | F = 0.832 (*p* = 0.438) | | | | |
| IL-8 | 1. Control | 38 | 11.97 | 1.78 | 3.92~66.33 | 0.042 | 0.004 |
| | 2. Small opacity | 67 | 15.42 | 1.56 | 7.66~48.31 | | 0.333 |
| | 3. Large opacity | 19 | 18.98 | 1.66 | 5.20~46.18 | | |
| | | | F = 6.009 (*p* = 0.003) | | | | |
| TNF-α | 1. Control | 38 | 3.57 | 1.29 | 2.29~6.98 | 0.286 | 0.024 |
| | 2. Small opacity | 67 | 3.94 | 1.33 | 2.02~10.95 | | 0.332 |
| | 3. Large opacity | 19 | 4.44 | 1.42 | 2.08~8.39 | | |
| | | | F = 3.768 (*p* = 0.026) | | | | |
| MCP-1 | 1. Control | 38 | 165.9 | 1.4 | 49.9~348 | 0.238 | 0.008 |
| | 2. Small opacity | 67 | 189.6 | 1.4 | 62.6~900 | | 0.162 |
| | 3. Large opacity | 19 | 228.7 | 1.6 | 118.7~900 | | |
| | | | F = 4.818 (*p* = 0.010) | | | | |

[1]GM: geometric mean, GSD: geometric standard deviation, unit: pg/ml
[2]p-values: calculated by ANOVA (Turkey's HSD) test
[3]Not detection: lower than limit of detection (LOD of IL-1β: 0.6 pg/ml)

## RESULTS

***General characteristics of the study populations.*** Number of study subjects by ILO categories of pneumoconiosis (ILO, 2002) was "small opacity" in 67 (54.1%), type in 40 (29.4%) and type II in 28 (20.6%), and "large opacity" in 19 (15.3%) (Table 1).

The characteristics of the study populations are showed in Table 2. The mean of age in the control (n = 38), the small opacity (n = 67), and the large opacity (n = 19) were 61.2 ± 7.2, 63.6 ± 7.7, and 66.0 ± 7.5, respectively. Body mass index (BMI), exposure period, pulmonary function, and smoking status did not show statistical difference between the control and pneumoconiosis groups.

***Concentration of blood cytokines according to general characteristics.*** The mean concentration of MCP-1 in low BMI (< 25 kg/m$^2$) was higher than that in high BMI (≥ 25 kg/m$^2$) ($p = 0.031$). There were no significant differences between levels of IL-1β, IL-6, IL-8, and TNF-α in blood and general characteristics such as age, exposure period, smoking status. Furthermore there were no significant differences between levels of measured cytokines in blood and criteria levels of %FVC, %FEV$_1$ and %FEV$_1$/FVC (Table 3).

***Concentration of blood cytokine according to ILO categories of pneumoconiosis.*** The mean concentrations of IL-8 ($p = 0.003$), TNF-α ($p = 0.026$), and MCP-1 ($p = 0.010$) in pneumoconiosis groups (small or large opacity) were higher than those of the control (Table 4). IL-8 levels in the small opacity, large opacity and the control were 15.42 pg/ml, 18.98 pg/ml, and 11.97 pg/ml, respectively. IL-8 levels in the small opacity ($p = 0.042$) and large opacity ($p = 0.004$) were higher than those of the control.

The mean concentrations of TNF-α (4.44 pg/ml) ($p = 0.024$) and MCP-1 (228.7 pg/ml) ($p = 0.008$) in the large opacity were higher than those of the control (3.57 pg/ml and 165.9 pg/ml, respectively). Although there were no statistical significance, TNF-α and MCP-1 levels in the small opacity tended to increase comparing with the control. For the IL-1β and IL-6 levels, there were no statistical significance between the control and pneumoconiosis groups.

The level of serum IL-8 in the severe group with the small opacity (ILO category II or III) was higher than that of the control (16.41 pg/ml *vs* 11.97 pg/ml, $p = 0.035$) (Table 5).

***Correlation between concentration of blood cytokine and associated variables.*** As shown in Table 6,

**Table 5.** Concentrations of blood cytokines according to x-ray profusion in pneumoconiosis patients with small opacity

| Cytokines | X-ray profusion[†] | N | GM[1] | GSD | Range | *p*-values of difference[2] | |
|---|---|---|---|---|---|---|---|
| | | | | | | 2 | 3 |
| IL-1β | 1. 0 | 38 | 1.24 | 3.16 | ND[3]~201.33 | 0.977 | 0.881 |
| | 2. I | 40 | 1.14 | 2.77 | ND~68.57 | | 0.953 |
| | 3. II, III | 27 | 1.09 | 2.60 | ND~116.66 | | |
| | | | F = 0.115 (*p* = 0.892) | | | | |
| IL-6 | 1. 0 | 38 | 1.09 | 2.63 | 0.36~17.41 | 0.632 | 0.619 |
| | 2. I | 40 | 1.33 | 2.99 | 0.38~193.09 | | 0.993 |
| | 3. II, III | 27 | 1.36 | 1.73 | 0.57~3.68 | | |
| | | | F = 0.586 (*p* = 0.558) | | | | |
| IL-8 | 1. 0 | 38 | 11.97 | 1.78 | 3.92~66.33 | 0.154 | 0.035 |
| | 2. I | 40 | 14.78 | 1.66 | 7.66~48.31 | | 0.674 |
| | 3. II, III | 27 | 16.41 | 1.41 | 9.08~32.26 | | |
| | | | F = 3.482 (*p* = 0.034) | | | | |
| TNF-α | 1. 0 | 38 | 3.57 | 1.29 | 2.29~6.98 | 0.276 | 0.317 |
| | 2. I | 40 | 3.93 | 1.35 | 2.23~10.95 | | 0.998 |
| | 3. II, III | 27 | 3.95 | 1.30 | 2.02~7.26 | | |
| | | | F = 1.539 (*p* = 0.219) | | | | |
| MCP-1 | 1. 0 | 38 | 165.9 | 1.4 | 49.9~348.0 | 0.322 | 0.174 |
| | 2. I | 40 | 186.2 | 1.5 | 86.7~900.0 | 0.868 | |
| | 3. II, III | 27 | 194.7 | 1.4 | 62.6~322.6 | | |
| | | | F = 1.864 (*p* = 0.160) | | | | |

[†]ILO Categories (0: 0/0, 0/1; I: 1/0, 1/1, 1/2; II: 2/1, 2/2, 2/3, III: 3/2)
[1]GM: geometric mean, GSD: geometric standard deviation, unit: pg/ml
[2]*p*-values: calculated by ANOVA (Turkey's HSD) test
[3]Not detection: lower than limit of detection (LOD of IL-1β: 0.6 pg/ml)

**Table 6.** Correlation coefficient between blood levels of cytokines concentration and independent variables

| | N | IL-1β | IL-6 | IL-8 | TNF-α | MCP-1 |
|---|---|---|---|---|---|---|
| Age (yrs)[1] | 124 | 0.005 | -0.152 | 0.048 | 0.076 | 0.011 |
| Exposure period (yrs)[1] | 124 | -0.101 | -0.141 | -0.038 | 0.103 | -0.090 |
| BMI ($kg/m^2$)[1] | 124 | -0.090 | -0.111 | -0.065 | -0.109 | -0.105 |
| Smoking habit (pack yrs)[1] | 124 | 0.221 | 0.070 | -0.028 | 0.001 | 0.030 |
| FVC, % predicted[1] | 124 | -0.043 | 0.036 | -0.019 | -0.126 | -0.103 |
| $FEV_1$, % predicted[1] | 124 | -0.140 | 0.047 | -0.095 | -0.106 | -0.169 |
| $FEV_1$/FVC ratio %[1] | 124 | -0.004 | 0.159 | -0.088 | -0.061 | -0.069 |
| X-ray Profusion[2a] | 105 | 0.218* | -0.004 | 0.224* | 0.306** | 0.213* |

All cases were Log transformed data
**p*<0.05, ***p*<0.01,
[1]Pearson's product moment correlation coefficient (*r*)
[2]Spearman's rank correlation coefficient (*rho*)
[a]Severity of pneumoconiosis with small opacity

**Table 7.** Correlation matrix of blood cytokine concentration (N = 124)

| | IL-1β | IL-6 | IL-8 | TNF-α | MCP-1 |
|---|---|---|---|---|---|
| IL-1β | 1.000 | | | | |
| IL-6 | 0.201* | 1.000 | | | |
| IL-8 | -0.071 | 0.048 | 1.000 | | |
| TNF-α | 0.188* | 0.229* | 0.206* | 1.000 | |
| MCP-1 | -0.005 | 0.300** | 0.255** | 0.116 | 1.000 |

All cases were Log transformed data
Pearson's product moment correlation coefficient
(*$p < 0.05$, **$p < 0.01$)

there was significant correlation between blood cytokines, including TNF-α (*rho* = 0.306, $p < 0.01$), IL-1β (*rho* = 0.218, $p < 0.05$), IL-8 (*rho* = 0.224, $p < 0.05$), and MCP-1 (*rho* = 0.213, $p < 0.01$), and pneumoconiosis severities in small opacity.

Correlation matrix among all cytokine levels was showed in Table 7. TNF-α correlated with IL-6 ($r$ = 0.229, $p < 0.01$) and IL-1β ($r$ = 0.188, $p < 0.05$), and IL-8 also correlated with MCP-1 ($r$ = 0.255, $p < 0.01$) and TNF-α ($r$ = 0.206, $p < 0.05$). MCP-1 correlated with IL-6

($r = 0.300$, $p < 0.01$).

## DISCUSSION

Toxicity and interaction of crystalline silica and coal dust are based on activation of macrophages and lung inflammation, many researchers have concerned about crucial mediators for the pulmonary disorder resulting from these mineral dust (Schins and Borm, 1999).

Inhaled dust leads to generation of ROS resulting from activated phagocytes in the lung, and transitional metals, including iron, copper, and vanadium were positively correlated with generation of ROS (Becker *et al.*, 1996; Castranova *et al.*, 1997; Dalal *et al.*, 1991; Shoemaker *et al.*, 1995; Tourmann and Kaufmann, 1994; Vallyathan, 2004; Wallaert *et al.*, 1990).

Inhaled inorganic particles cause release of cytokines resulting in inflammation and injury to lung epithelial cell (Lasky *et al.*, 2005). Recruitment of inflammatory cells such as monocytes, macrophages, and neutrophils plays an important role in inflammatory process in the lung. Inflammation and its progression may depend upon dust concentration and it is proceeded after discontinuation of exposure (Donaldson *et al.*, 1990). Chronic injury involving loss of phagocytosis may continuously lead to inflammation with persistent oxidative stress (Perlman *et al.*, 2005).

TNF-$\alpha$ and IL-1 are early response mediators of lung inflammation and released by activated macrophages. They are initiators of cytokine networks and lead to neutrophil recruitment and chemotaxis (Lukacs and Ward, 1996). TNF-$\alpha$ and IL-1 play various actions including synergistic effects in inflammatory and immune response (Gulumian *et al.*, 2006). There are two forms, IL-1$\alpha$ and IL-1$\beta$, which play same role *in vitro*. IL-1 released from phagocytes, polynuclear white blood cells, and fibroblast plays an important role in lung fibrosis (Kolb *et al.*, 2001). TNF-$\alpha$ would be responsible for the initiation and perpetuation of the inflammatory reaction observed in the lung of patients with PMF. TNF-$\alpha$, which can directly induce fibroblast proliferation, could also trigger the production of mediators (Vanhee *et al.*, 1995). Blood levels of TNF-$\alpha$ and IL-1 were tended to increase in CWP (Vallyathan *et al.*, 2000). Schins and Borm (1995) reported that TNF-$\alpha$ was a predicted biomarker for progressive pneumoconiosis and its level correlated with severity of pneumoconiosis. Gulumian *et al.* (2006) reported that TNF-$\alpha$ was useful index for coal dust exposure and was useful biomarker for pneumoconiosis with progressive fibrosis in the lung. In this study, we found that the level of IL-1$\beta$ was not increased in the subjects with pneumoconiosis but positively correlated with x-ray profusion in pneumoconiosis patients with small opacity ($p < 0.05$). The level of TNF-$\alpha$ was higher in pneumoconiosis patients with large opacity than the control ($p < 0.05$), and positively correlated with x-ray profusion in the subjects with small opacity ($p < 0.01$). Theses results are in agreement with those of previous studies.

The level of IL-6 was increased in the patients with asthma and lung fibrosis (Reuben *et al.*, 2004). The level of IL-6 was increased in BALF or alveolar macrophages in CWP and associated with disease progression (Gosset *et al.*, 1991; Vallyathan *et al.*, 2000). Zhai *et al.* (2002) reported that the level of serum IL-6 correlated with pneumoconiosis classifications. In this study, the level of IL-6 tended to increase in the subjects with pneumoconiosis compared with the control but there was no significant difference ($p > 0.05$).

IL-8 is a structurally similar family of cytokine called chemokine, which demonstrates chemotactic activity for neutrophils. IL-8 is produced in response to proinflammatory stimuli. The accumulation of inflammatory leukocytes in the lung is hallmark of either acute or chronic pulmonary inflammation (Strieter *et al.*, 1993). The level of IL-8 was higher in the pneumoconiosis patients with small ($p < 0.05$) and large opacity ($p < 0.01$) than the control, and positively correlated with x-ray profusion in the subjects with small opacity ($p < 0.01$). The level of IL-8 in the severe group with the small opacity (ILO category II or III) was higher than that of the control ($p < 0.05$).

Many researchers have studied the relationship between proinflammatory cytokines, TNF-$\alpha$ and IL-1, and crystalline silica because TNF-$\alpha$ and IL-1 activate releasing of IL-6 or IL-8 (Gulumian *et al.*, 2006). In this study, TNF-$\alpha$ correlated with IL-6 ($r = 0.229$, $p < 0.01$), IL-8 ($r = 0.206$, $p < 0.05$), and IL-1$\beta$ ($r = 0.188$, $p < 0.05$).

MCP-1 plays an important role in the initial recruitment of cells such as lymphocytes and a small number of monocytes, and main role is as an activator and chemattractant of monocytes, leukocytes or lymphocytes (Toews, 2000). MCP-1 has been implicated in a variety of inflammatory diseases such as alveolitis and idiopathic pulmonary fibrosis (Loetscher *et al.*, 1994; Yoshimura *et al.*, 1989). Boitelle *et al.* (1997) reported that the level of MCP-1 was increased in BALF of CWP. MCP-1 regulates IL-1 and IL-6 (Biswas and Sodhi, 2002). In this study, the level of MCP-1 was higher in pneumoconiosis patients with large opacity than the control ($p < 0.05$), and positively correlated with x-ray profusion in the subjects with small opacity ($p < 0.01$). MCP-1 correlated with IL-6 ($r = 0.300$, $p < 0.01$) and IL-8 ($r = 0.255$, $p < 0.01$).

Although pneumoconiosis is the most prevalent lung disease showing decreasing of pulmonary function and emphysema (Schins and Borm, 1999), we found that measured cytokines were not correlated with the results of PFT. The reason of theses results can be explained that decreased PFT is the result of inflammation or fibrosis in the lung but cytokines are effects on current response of inflammation. Other possibilities may include that PFT is affected by the difference in the anatomy of the respiratory tract such as restriction of bronchus.

In accordance with previous studies, the level of blood cytokines showed significant difference among the categories of pneumoconiosis according to radiological findings, but the difference was not clear. For these reasons, although we discarded the subjects with the inflammation findings of liver and kidney in this study, the diagnostic specificity of measured blood cytokines is not satisfactory in the lung, because the levels of blood cytokines are affected by almost inflammatory response. It was necessary to monitor for the effects between cytokines and pneumoconiosis progression, including decreasing PFT and exacerbation of radiological findings. Future studies will be required to ascertain the cytokine profile that is present in pneumoconiosis patient using lung specific specimens such as BALF, exhaled breath condensate, or lung tissue.

## ACKNOWLEDGEMENT

This study was conducted by the financial contribution of Korea Workers Accident Medical Corporation (KWAMCO).
